# Fate of engineered nanomaterials at the human epithelial lung tissue barrier *in vitro* after single and repeated exposures

**DOI:** 10.3389/ftox.2022.918633

**Published:** 2022-09-16

**Authors:** Roman Lehner, Ilaria Zanoni, Anne Banuscher, Anna Luisa Costa, Barbara Rothen-Rutishauser

**Affiliations:** ^1^ Adolphe Merkle Institute, University of Fribourg, Fribourg, Switzerland; ^2^ CNR-ISTEC-National Research Council of Italy, Institute of Science and Technology for Ceramics, Faenza, Ravenna, Italy

**Keywords:** lung epithelial tissue, fate, single and repeated exposures, engineered nanomaterials, cellular fraction

## Abstract

The understanding of the engineered nanomaterials (NMs) potential interaction with tissue barriers is important to predict their accumulation in cells. Herein, the fate, e.g., cellular uptake/adsorption at the cell membrane and translocation, of NMs with different physico-chemical properties across an A549 lung epithelial tissue barrier, cultured on permeable transwell inserts, were evaluated. We assessed the fate of five different NMs, known to be partially soluble, bio-persistent passive and bio-persistent active. Single exposure measurements using 100 µg/ml were performed for barium sulfate (BaSO_4_), cerium dioxide (CeO_2_), titanium dioxide (TiO_2_), and zinc oxide (ZnO) NMs and non-nanosized crystalline silica (DQ_12_). Elemental distribution of the materials in different compartments was measured after 24 and 80 h, e.g., apical, apical wash, intracellular and basal, using inductively coupled plasma optical emission spectrometry. BaSO_4_, CeO_2_, and TiO_2_ were mainly detected in the apical and apical wash fraction, whereas for ZnO a significant fraction was detected in the basal compartment. For DQ_12_ the major fraction was found intracellularly. The content in the cellular fraction decreased from 24 to 80 h incubation for all materials. Repeated exposure measurements were performed exposing the cells on four subsequent days to 25 µg/ml. After 80 h BaSO_4_, CeO_2_, and TiO_2_ NMs were again mainly detected in the apical fraction, ZnO NMs in the apical and basal fraction, while for DQ_12_ a significant concentration was measured in the cell fraction. Interestingly the cellular fraction was in a similar range for both exposure scenarios with one exception, i.e., ZnO NMs, suggesting a potential different behavior for this material under single exposure and repeated exposure conditions. However, we observed for all the NMs, a decrease of the amount detected in the cellular fraction within time, indicating NMs loss by cell division, exocytosis and/or possible dissolution in lysosomes. Overall, the distribution of NMs in the compartments investigated depends on their composition, as for inert and stable NMs the major fraction was detected in the apical and apical wash fraction, whereas for partially soluble NMs apical and basal fractions were almost similar and DQ_12_ could mainly be found in the cellular fraction.

## Introduction

Over the past years, the increase in engineered nanomaterial (NMs) research has been concomitant with the overwhelming increase in nanotechnology related products being produced (Gwinn and Vallyathan, 2006; Maynard et al., 2006). This new industrial revolution promises to provide advantages for numerous applications, including medicine, consumer products (e.g., food additives, cosmetics, sporting equipment), environmental remediation and information technology (Maynard et al., 2006). To realize these proposed benefits, heightened research has been performed to determine if the potential benefits of nanotechnology can be utilized without adverse effects on humans. Thus, a better understanding of the consequences upon human exposure to these NMs is a prerequisite for their safe-by-design and the successful use in applications (Bastus and Puntes, 2018).

The potential hazards of NMs are not only determined by the physico-chemical properties of the particle per se (Moore et al., 2015; Gao and Lowry, 2018), but also on the interactions of the particles with immediate surrounding environments (Ostrowski et al., 2015). Once inside cells, NMs distribution in different compartments and possible dissolution also provides indications as to their potential biological impact upon occupational and end-user exposure, as well as how to specifically design NMs for effective cell targeting and drug delivery.

There is a need to apply mechanistic based testing strategies enabling to understand interactions of NMs at a cellular level. It is accepted that *in vitro* results can be useful for ranking NMs either by enabling a deeper insight into mechanisms of NMs-induced (potentially even nano-specific) effects or by serving as a basis for follow-up *in vivo* studies (Kroll et al., 2009; Park et al., 2009; Drasler et al., 2017). So far, the majority of publications concerning NM effects at the cellular level focus upon acute effects and usually at high concentrations (Krug and Wick, 2011; Krug, 2018; Leibrock et al., 2020). However, occupational exposure is assumed to occur over a prolonged period of time and the experimental design should be optimized to use relevant concentrations (Gangwal et al., 2011; Bessa et al., 2020). The cellular effects that occur over a prolonged period can only be measured by post-exposure incubation of the exposed cells for extended (i.e., days-to-weeks) durations. Few studies describe chronic exposure scenarios and assess toxic effects. For instance, a chronic exposure scenario using the alveolar type II epithelial cell line A549 in suspension has been described (Thurnherr et al., 2010). In this study, the long-term effects of industrial multi-walled carbon nanotubes were compared to acute effects in lung cells (in suspension) experiments. The sub-lethal adverse effects were found to be elicited by acute carbon nanotube exposure, however, the long-term presence (over 6 months) and accumulation of this material did not have any major impact on cell viability or functionality. Another study also described the use of the BEAS-2B cell line (human bronchial epithelial cells) to assess long-term exposure in suspension (i.e., 1 month), to low doses of cigarette smoke. It was observed that the chronic exposure lead to a change in the cell phenotype and to novel gene expressions (Veljkovic et al., 2011).

The interaction of NM with the epithelial tissue in an organism is a key aspect of assessing hazard (Rothen-Rutishauser et al., 2012; Hamm et al., 2017). Once NMs are inside the human body, they interact with the cell surface and can be internalized by the cells. Different uptake mechanisms have been identified (Sousa de Almeida et al., 2021) and a cascade of events were described as part of Adverse Outcome Pathways (AOPs) (Halappanavar et al., 2020) that lead to various adverse outcomes (Manke et al., 2013). NMs can interact with proteins, but also membranes, cells, DNA, and organelles, establishing a series of NMs/biological interfaces that depend on colloidal NM properties (Z potential, particle size distribution, etc.) as well as dynamic bio-physicochemical interactions (formation of bio-corona, settling rates, etc.) (Langevin et al., 2018; Chetwynd and Lynch, 2020; Precupas et al., 2020). At the same time nano-bio interactions can cause an increase in the cellular oxidative stress, generating excessive levels of reactive oxygen species (ROS) and possibly causing adverse effects, from inflammation to cell damage and death. Cell culture medium interaction with NMs could also promote the leaching of ions that interact directly with cell membranes (Zanoni et al., 2022). Finally, NMs can be taken up by cells and act as “Trojan horse”, so carry and release other, toxic, chemicals that are attached to its surface into cells (Limbach et al., 2007; Monopoli et al., 2011).

In the H2020 funded PATROLS project (Physiologically Anchored Tools for Realistic nanOmateriaL hazard aSsessment), we assessed the fate of five different NMs, grouped within the DF4nanoGrouping framework (Landsiedel et al., 2017) as partially soluble (MG1), bio-persistent passive (MG3) and bio-persistent active (MG4) groups. We used the already widely investigated NMs belonging to the JRC repository list (Singh et al., 2012; Rasmussen et al., 2013; Landsiedel et al., 2014; Singh et al., 2014; OECD, 2018): zinc oxide (MG1), barium sulfate (MG3), cerium dioxide, titanium dioxide and the control non-nanosized material crystalline silica (DQ_12_), belonging to MG4. We studied the fate of NMs in epithelial cells cultured on permeable transwell inserts. A549 cells representing human alveolar type II like cells were used (Lieber et al., 1976), as they are well characterized and widely used as *in vitro* lung epithelial cell models (Foster et al., 1998). The A549 cell line has proven useful for studying cytotoxicity, oxidative stress, and/or the (pro-)inflammatory response following exposure to various (nano)materials (dos et al., 2011; Martin and Sarkar, 2017; Bisig et al., 2019). More complex co-cultures have been described in the literature to study the toxicity or biodistribution of (nano)materials, e.g., A549 cells combined with THP-1 macrophages (Arezki et al., 2021; Meindl et al., 2021), or A549 cells combined with human-monocyte derived macrophages and dendritic cells (Chortarea et al., 2018). Because we found no significant different biodistribution of 18-nm gold nanoparticles in A549 cells alone compared with A549 cells co-cultured with macrophages and dendritic cells in a previous study (Bachler et al., 2015), we decided to focus only the mono-culture system as the main objective of our work was to compare single with repeated exposure and to relate the fractional distribution to the physico-chemical properties of the NMs used. We compared the elemental distribution of NMs across different fractions using A549 epithelial tissues, i.e., apical compartment, apical wash, intracellularly and in the basal compartments, by inductively coupled plasma optical emission spectrometry (ICP-OES). We verified if a possible accumulation over repeated exposures might result in similar material distributions in the various fractions as upon one exposure to high concentrations by performing single NM exposure with 100 μg/ml and incubation for 24 and 80 h and compared with repeated exposures, 4 × 25 μg/ml, and sample preparation at 24, 48, 72, and 80 h.

## Materials and methods

### Cell culture

The human adenocarcinomic alveolar basal epithelial type II cell line A549 was obtained from American Type Culture Collection (ATCC, CCL-185TM). Cells were cultivated at 37°C under a 5% CO_2_ water saturated atmosphere in complete medium consisting of Roswell Park Memorial Institute medium (RPMI 1640) (Gibco) supplemented with 10% heat inactivated fetal bovine serum (FBS) (Gibco), 1% L-glutamine (Gibco), and 1% penicillin and streptomycin (Gibco). The cells were cultured as described (Bisig et al., 2019). Briefly, A549 cells were grown in 75 cm^2^ flasks (Corning, United States) and sub-cultured once per week at a splitting ratio of 1:12. A549 cells were cultured at a density of 2.5 × 10^5^ cells per insert on BD Falcon cell culture inserts (high pore density PET membranes, 3.0 μm pore size) under submerged conditions for 5 days prior to exposure without cell media change as described previously (Bisig et al., 2019).

### Materials and sample preparation

NMs including titanium dioxide (TiO_2_) JRCNM01005a (former NM-105), and zinc oxide (ZnO) JRCNM01101a (former NM-111), were provided as nano-powders by JRC in Ispra (Italy). Barium sulphate (BaSO_4_) NM-220 and cerium dioxide (CeO_2_) NM-212 were purchased from Fraunhofer-Gesellschaft (Germany). Crystalline α-quartz-silica, DQ_12_ (Robock, 1973), was provided by Institute of Occupational Medicine (IOM). The dissolved ionic Ba, Ce, Si, Ti, and Zn standard for ICP-OES, hydrogen peroxide solution (95321) and nitric acid (84380-M) were purchased from Sigma-Aldrich (St. Louis, MO, US). Samples were prepared starting from stocks at 2.56 mg/ml in MilliQ water + 0.05% w/w bovine serum albumin (BSA), prepared accordingly to the NANoREG dispersion protocol (Jensen, 2014). The stocks were then diluted in RPMI + 10% FBS medium reaching the desired concentration of 25 or 100 µg/ml.

### Material characterization

Hydrodynamic diameter and Zeta Potential of the nano-suspensions both in MilliQ water and in RPMI + 10% FBS medium were determined by dynamic light scattering (DLS) and electrophoretic light scattering (ELS) measurements, respectively using a Zetasizer Nano instrument ZSP (model ZEN5600, Malvern Instruments, United Kingdom). The samples were diluted at 100 μg/ml. The size distribution and zeta potential data, with relative standard deviation (rsd %), of all samples were obtained by averaging the triplicates and three repetitions (*n* = 3).

### Transmission electron microscopy

For transmission electron microscopy (TEM), diluted samples (100 μg/ml) were dropcasted onto 300 mesh carbon membrane-coated copper grids following a procedure described elsewhere (Michen et al., 2015). TEM experiments were carried out on a FEI Tecnai Spirit operating at a voltage of 120 kV and equipped with a side-mounted Veleta CCD camera (Olympus).

### Single- and repeated cell exposure scenarios and sample collection

**FIGURE 1:**
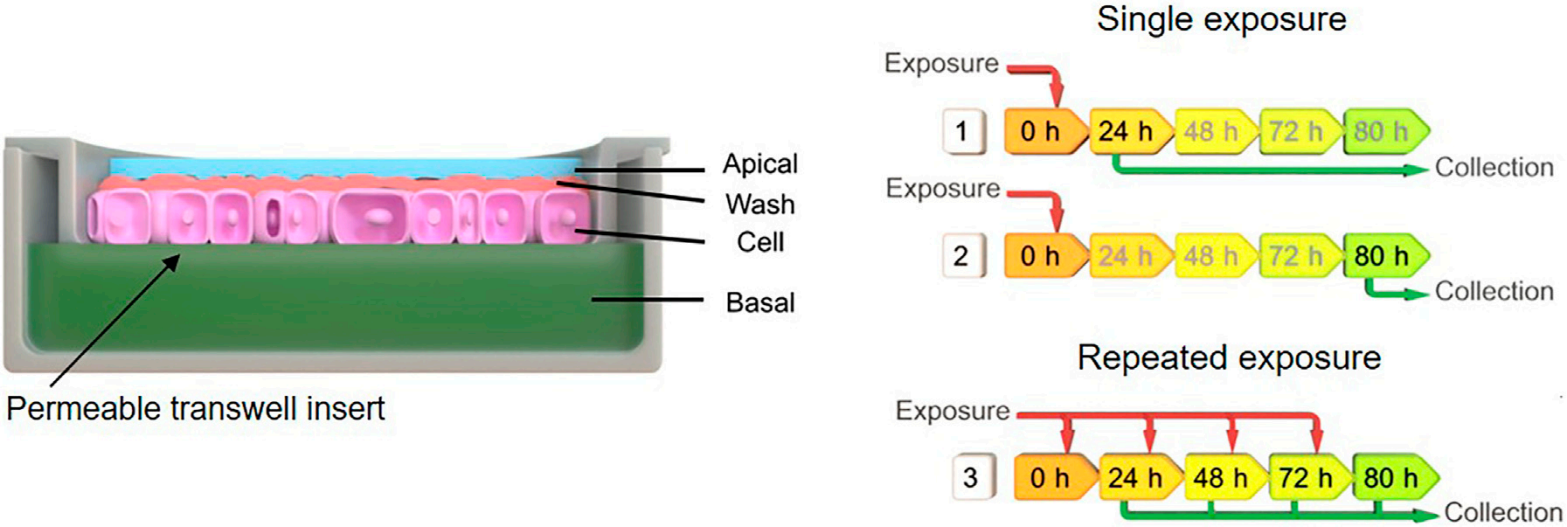
Schematic indicating the exposure scenarios and sample collection after material exposures to A549 monolayer. Exposure scenario one implies single exposure (100 µg/ml) at time point zero and sample collection after 24 h, for scenario two single exposure (100 µg/ml) at time point zero and sample collection after 80 h, and scenario three repeated exposure (25 µg/ml) and multiple collection after 24, 48, 72, and 80 h.

Single exposure of ZnO NMs, BaSO_4_ NMs, CeO_2_NMs, DQ_12_, and TiO_2_ NMs were done with a concentration of 100 µg/ml added to the apical side and samples were collected after 24 and 80 h. In the long-term repeated exposures on A549 cells, the cells were exposed repeatedly from the apical side every day up to 5 days, using 25 µg/ml NMs concentration, resulting in a total exposure of 100 µg/ml and 5 days incubation time in total. The medium volume on the apical side was 0.5 ml and on the basal side 1.5 ml. Apical, apical cell wash and basal samples were collected after 24, 48, 72, and 80 h of exposure, while the cell fraction was collected at the end of the experiment at 80 h. A schematic overview depicted in Figure 1 represents the experimental design. Apical and basal fractions were directly collected while for the apical wash fraction, cells were washed three times with PBS to remove loosely adhered materials and all wash steps were pooled. To analyse the cellular uptake and strong adsorption of the material at the cellular membrane, whole membranes with the cells attached were cut out. All measurements were done in triplicates and three biological repetitions (*n* = 3).

### Cell viability assay

A possible cytotoxic effect of the different materials on the A549 cell monolayer were investigated by the release of lactate dehydrogenase (LDH) into the supernatant (apical and basal fractions) as a result of cell membrane rupture. LDH release was evaluated using a commercially available LDH diagnostic kit (Roche Applied Science, Germany), according to the manufacturer’s protocol. Each material was tested in triplicate and the enzyme activity was measured photometrically at an absorbance of 490 nm with reference wavelength of 630 nm. As a positive control 0.2% Triton-X-100 was applied apically. LDH values are presented relative to the negative control (untreated cells).

### Inductively coupled plasma optical emission spectrometry

The collected samples were acid digested and elemental distribution of the materials in different *in vitro* test compartments measured by inductively coupled plasma optical emission spectrometry using an ICP-OES 5100—vertical dual view apparatus coupled with OneNeb nebulizer (Agilent Technologies, Santa Clara, CA, United States). Digestive procedure was performed adding 0.2 ml of hydrogen peroxide (H_2_O_2_ 30 wt % in water), 0.2 ml of sulfuric acid (H_2_SO_4_ 96%), 0.2 ml of phosphoric acid (H_3_PO_4_ 85%) and 0.2 ml of nitric acid (HNO_3_ 65%) in to 0.5 ml sample and adding 1.5 ml of MilliQ water. The treated samples were ultrasonicated for 10 min in an ultrasonic bath. Calibration curves were obtained with 0.01, 0.05, 0.1, 1.0, 10.0, and 50.0 µg/ml standards prepared in RPMI, using the same digestive procedure applied to samples. Standards were treated adding a 10% v/v of sulfuric acid, phosphoric acid and of nitric acid, and ultrasonicated for 10 min, as well.

### Hyperspectral imaging

To investigate particle interactions by A549 cells, dark-field imaging was performed for cells incubated with 100 μg/ml for 80 h. The cells were washed three times with PBS and fixed with 4% PFA in PBS for 10 min at room temperature. The fixed cells stored in PBS were then removed from the membranes of 12-well inserts using a cell scraper, transferred into an Eppendorf tube, diluted, and washed with 100 µl PBS (Gibco). 10 µl of this mixture was then combined with 30 µl of mounting media (Fluoromount™ Aqueous Mounting Medium, F4680, Sigma-Aldrich). Finally, 20 µl were mounted onto a microscopy glass slide that was previously cleaned for 10 min in 70% ethanol in ultrasonic bath and dried by compressed air. After 24 h for hardening, the samples were visualized by ×100 magnification with numerical aperture 0.8 in an enhanced darkfield microscopy setup (Cytoviva Inc., Auburn, United States). The hyperspectral data cube was achieved using a spectrophotometer (400–1000 nm) and recorded on a Pixelfly camera (PCO AG, Kelheim, Germany) using a quartz halogen aluminum reflector light source at an exposure time of 250 ms per line (720 lines).

### Statistical analysis

All data are presented as mean ± standard deviation. A total of three independent experiments (*n* = 3) were performed for the LDH and ICP-OES measurements. Statistical analysis was performed using GraphPad Prism (GraphPad Software Inc., La Jolla, United States). Assuming normal distribution of the data sets, a parametric one-way analysis of variance (ANOVA) was performed, followed by Dunnett’s multiple comparison test. In all experiments, results were considered significant if *p* < 0.05.

## Results

### Material characterization

**FIGURE 2:**
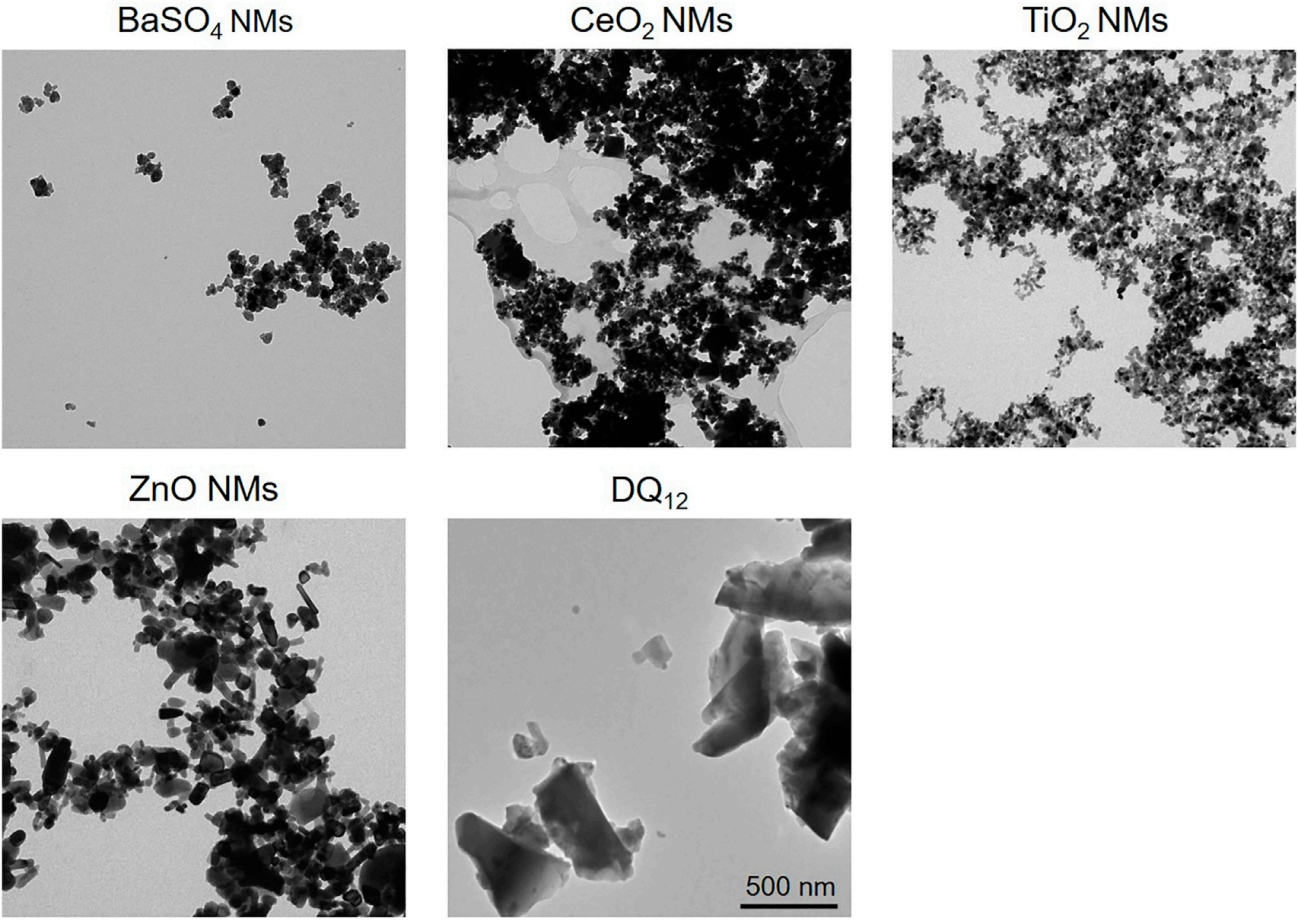
TEM images of BaSO_4_ NMs (D_mean_ = 32 nm), CeO_2_ NMs (D_mean_ = 14 nm), TiO_2_ NMs (Dmean = 25 nm), ZnO NMs (D_mean_ = 41 nm), and crystalline silica DQ_12_ (D_min_ = 200 ± 30 nm, D_max_ = 300 ± 50 nm). Scale bar is 500 nm for all images.

In order to characterize the colloidal behavior of the materials used, we evaluated the hydrodynamic diameter and the zeta potential of ZnO NMs, BaSO_4_ NMs, CeO_2_ NMs, DQ_12_, and TiO_2_ NMs in the testing media, i.e., MilliQ + 0.05% BSA, and the complete cell culture medium, i.e., RPMI + 10% FBS, at a concentration of 100 μg/ml (Table 1). This characterization aims to identify possible aggregation of the particles that can occur in cell culture medium used for cell growth, influencing the behavior and sub-sequent cell uptake. Data reported in Table 1, shows the same degree of agglomeration and comparable negative zeta potential for all samples diluted in cell culture medium and in MilliQ water for comparison, due to the presence of BSA surface coating (protein corona formation), deriving by stock dispersion preparation protocol. The sample that results almost monodispersed is crystalline silica whose hydrodynamic diameter is comparable with that observed by TEM (Figure 2). This is explained by fact that silica, being an acidic oxide, shows a natural high negative zeta potential when dispersed in water, keeping its negative zeta potential charge also once coated with negatively charged BSA, added to stock suspension.

**TABLE 1:** DLS and ELS measurements of NM stock solutions prepared in MilliQ + BSA and diluted in MilliQ water and RPMI + 10% FBS media at 100 μg/ml.

Materials	MilliQ medium				RPMI +10% FBS medium			
	pH	Size (nm)	PDI	Zeta potential (mV)	pH	Size (nm)	PDI	Zeta potential (mV)
ZnONMs	6.1	360 ± 4	0.200	−16.7 ± 0.5	7.1	418 ± 8	0.200	−10.0 ± 1.3
BaSO_4_ NMs	5.5	296 ± 16	0.450	−18.6 ± 0.9	7.2	274 ± 54	0.500	−10.5 ± 0.9
CeO_2_ NMs	6.0	346 ± 29	0.300	−19.5 ± 7.2	7.1	399 ± 37	0.300	−9.0 ± 0.7
DQ_12_	6.0	475 ± 45	0.350	−29.1 ± 6.6	7.2	467 ± 47	0.600	−9.9 ± 0.6
TiO_2_ NMs	6.1	284 ± 19	0.200	−17.2 ± 1.7	7.1	298 ± 5	0.200	−10.2 ± 0.8

### Cell viability

**FIGURE 3:**
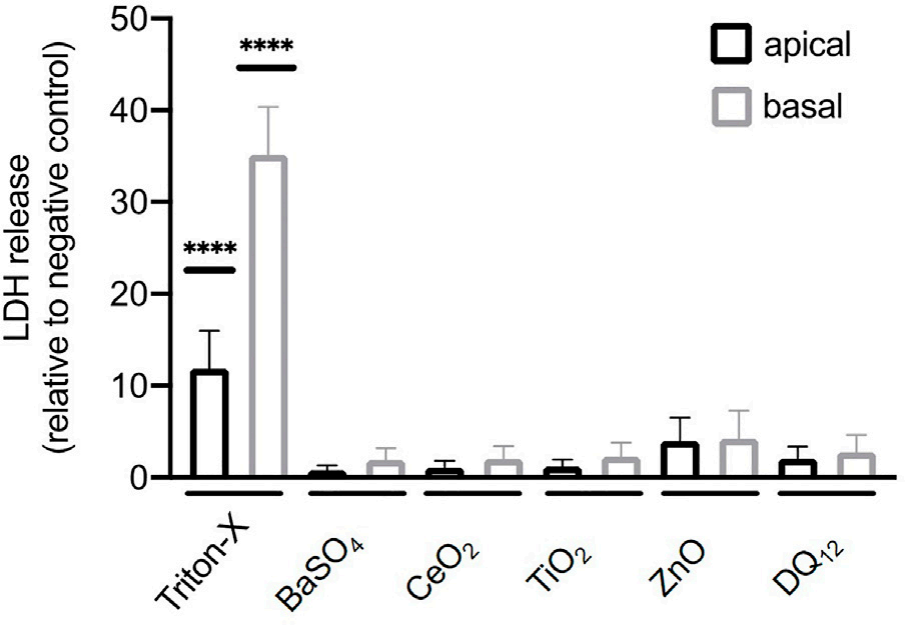
Cytotoxicity determined by measuring the release of lactate dehydrogenase (LDH) by A549 cells after the exposure of 100 µg/ml BaSO_4_NMs, CeO_2_NMs, TiO_2_NMs, ZnONMs, and DQ_12_ for 80 h. A total of three independent experiments (*n* = 3), consisting of three single replicates each, were performed. Statistically significant difference to the negative control is denoted by asterisks, where **** is *p* < 0.0001.

The cytotoxicity of all materials was assessed in cells exposed to 100 µg/ml of ZnO NMs, BaSO_4_ NMs, CeO_2_ NMs, TiO_2_ NMs, and DQ_12_ for 80 h *via* quantification of LDH released into the apical and basal cell culture medium. No significant cytotoxicity (*p* > 0.05) was observed after 80 h following exposure compared to untreated cells. A significant LDH increase could only be shown for the positive control (Triton-X-100) (Figure 3).

### Interaction of NMs with cells

**FIGURE 4:**
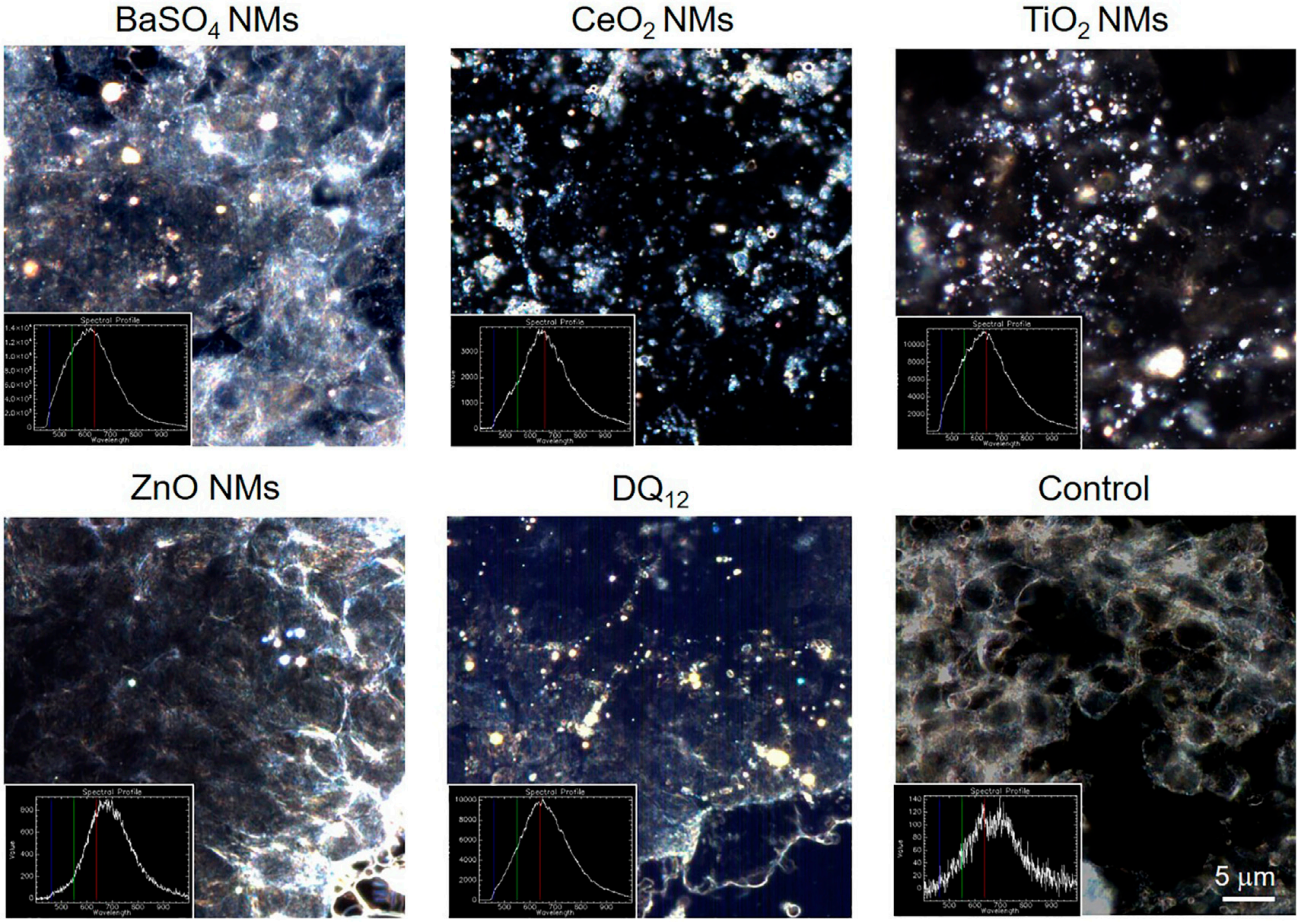
Hyperspectral images of NMs and DQ_12_ only and of A549 epithelial cells treated with 100 µg/ml for 80 h and the corresponding spectra. Please note that for some images the focus of the materials and cells do not match and therefore images may look blurry. Scale bar is 5 μm for all images.

Enhanced hyperspectral imaging (HSI) allows imaging of cells and materials as well as the spectral confirmation of the materials. Images with the corresponding spectra are depicted in Figure 4. The results show that after exposure to 100 μg/ml for 80 h all NMs could be detected associated with A549 cells but the amounts varied between NMs. On a qualitative basis, most intracellular NMs were observed after exposure to CeO_2_ NMs, DQ_12_ and TiO_2_ NMs followed by BaSO_4_ NMs. Only few ZnO NMs particles could be detected in A549 cells which could be attributed to the increased solubility of ZnO NMs in cell culture medium and once internalized by cells, as reported in a previous study (Zanoni et al., 2022). It is important to mention that it was not possible to clearly distinguish between intracellular particles or those that are membrane-associated with this analysis.

### Cell exposures and elemental distribution in different *in vitro* test compartments

**FIGURE 5:**
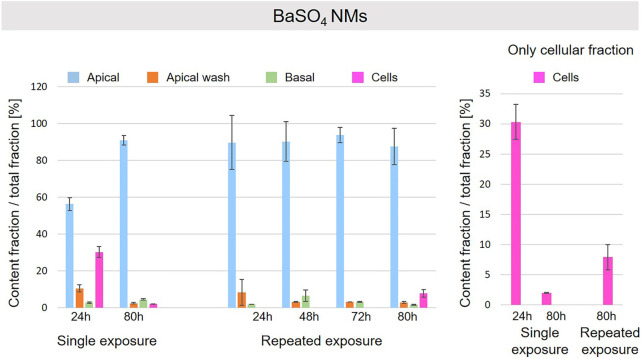
Fate of BaSO_4_ NMs at the A549 tissue barrier after single and repeated exposures. For single exposures BaSO_4_ NMs was exposed at a concentration of 100 µg/ml and the different fractions were collected after incubation for 24 and 80 h. For repeated exposures the cells were exposed repeatedly every day up to 80 h, using 25 µg/ml BaSO_4_ NMs concentration, resulting in a total exposure of 100 µg/ml and 80 h incubation time in total. Apical, apical cell wash and basal samples were collected after 24, 48, 72, and 80 h of exposure. Right side: This graph only depicts the cellular fraction. A total of three independent experiments (*n* = 3), with three single replicates each, were performed.

**FIGURE 6:**
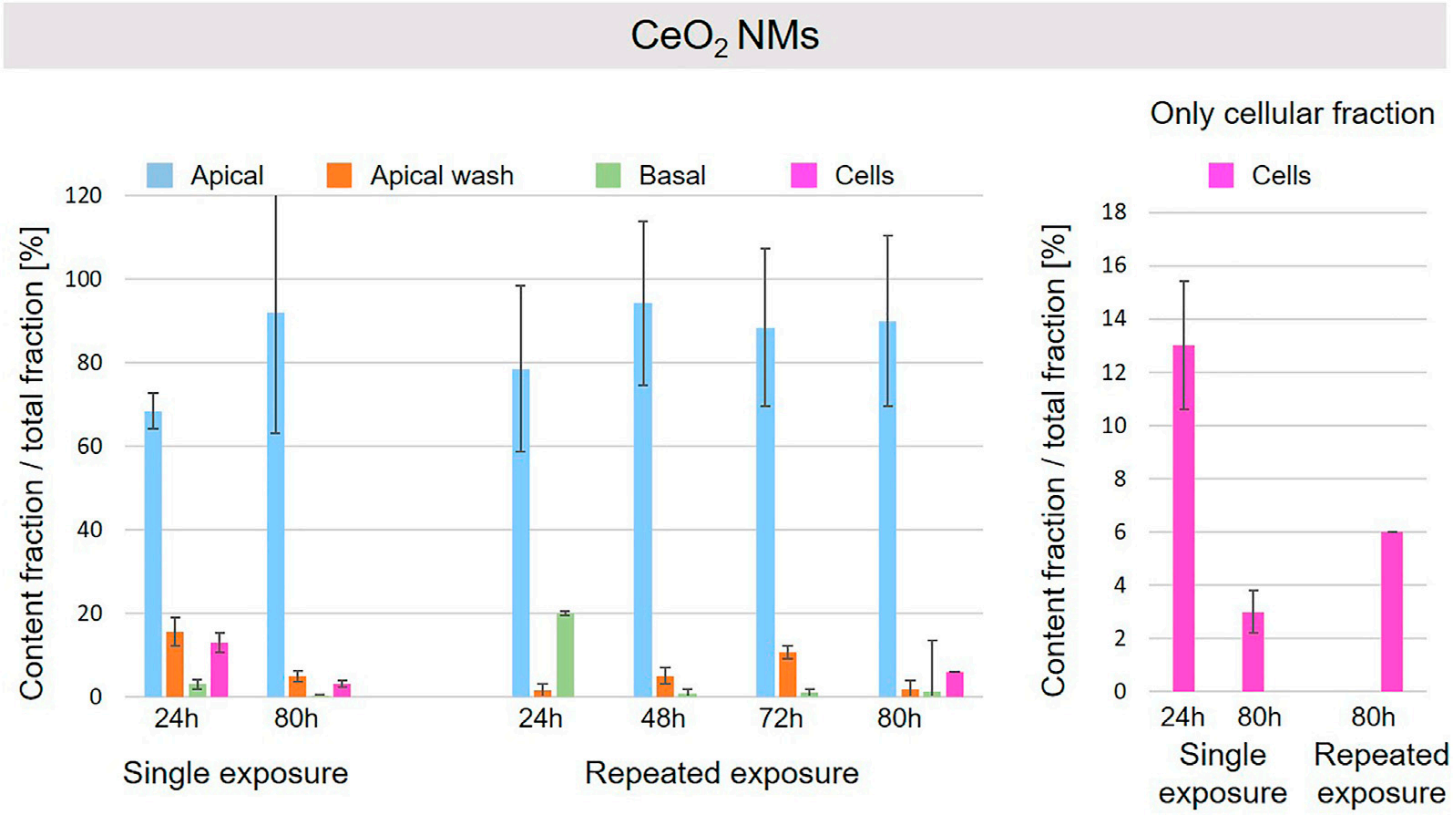
Fate of CeO_2_ NMs at the A549 tissue barrier after single and repeated exposures. For single exposures CeO_2_ NMs was exposed at a concentration of 100 µg/ml and the different fractions were collected after incubation for 24 and 80 h. For repeated exposures the cells were exposed repeatedly every day up to 80 h, using 25 µg/ml CeO_2_ NMs concentration, resulting in a total exposure of 100 µg/ml and 80 h incubation time in total. Apical, apical cell wash and basal samples were collected after 24, 48, 72, and 80 h of exposure. Right side: This graph only depicts the cellular fraction. A total of three independent experiments (*n* = 3), with three single replicates each, were performed.

**FIGURE 7:**
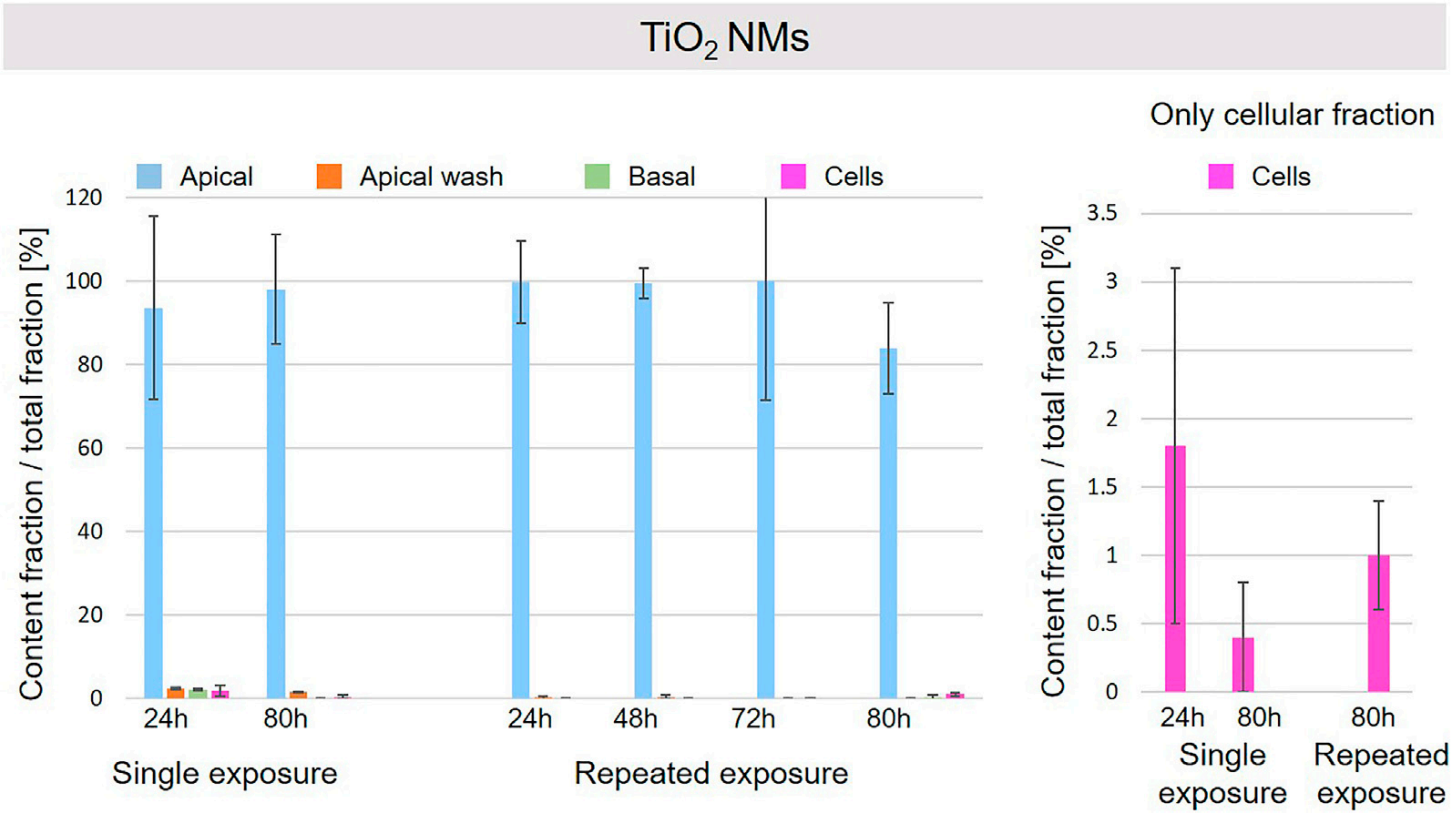
Fate of TiO_2_ NMs at the A549 tissue barrier after single and repeated exposures. For single exposures TiO_2_ NMs was exposed at a concentration of 100 µg/ml and the different fractions were collected after incubation for 24 and 80 h. For repeated exposures the cells were exposed repeatedly every day up to 80 h, using 25 µg/ml TiO_2_ NMs concentration, resulting in a total exposure of 100 µg/ml and 80 h incubation time in total. Apical, apical cell wash and basal samples were collected after 24, 48, 72, and 80 h of exposure. Right side: This graph only depicts the cellular fraction. A total of three independent experiments (*n* = 3), with three single replicates each, were performed.

**FIGURE 8:**
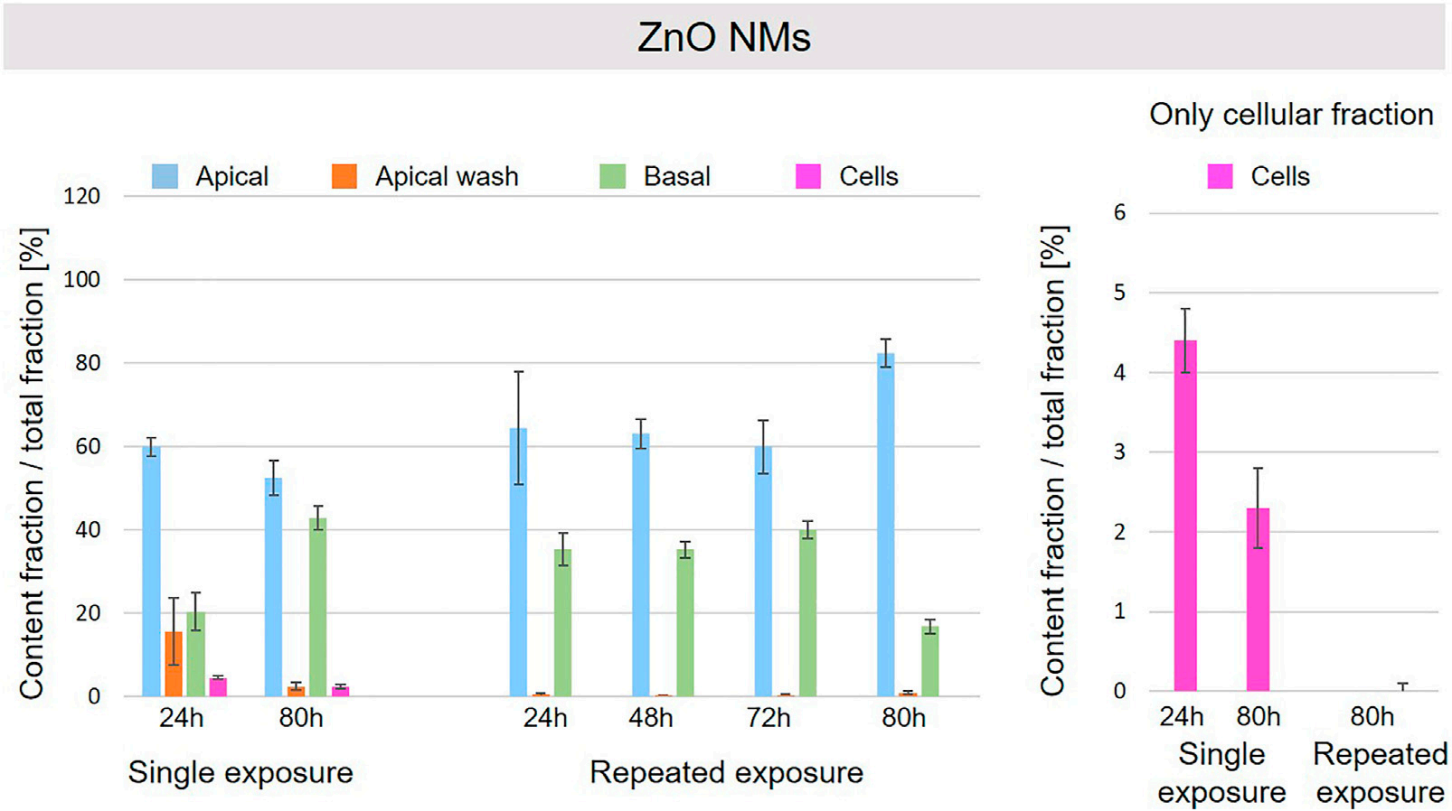
Fate of ZnO NMs at the A549 tissue barrier after single and repeated exposures. For single exposures ZnO NMs was exposed at a concentration of 100 µg/ml and the different fractions were collected after incubation for 24 and 80 h. For repeated exposures the cells were exposed repeatedly every day up to 80 h, using 25 µg/ml ZnO NMs concentration, resulting in a total exposure of 100 µg/ml and 80 h incubation time in total. Apical, apical cell wash and basal samples were collected after 24, 48, 72, and 80 h of exposure. Right side: This graph only depicts the cellular fraction. A total of three independent experiments (*n* = 3), with three single replicates each, were performed.

**FIGURE 9:**
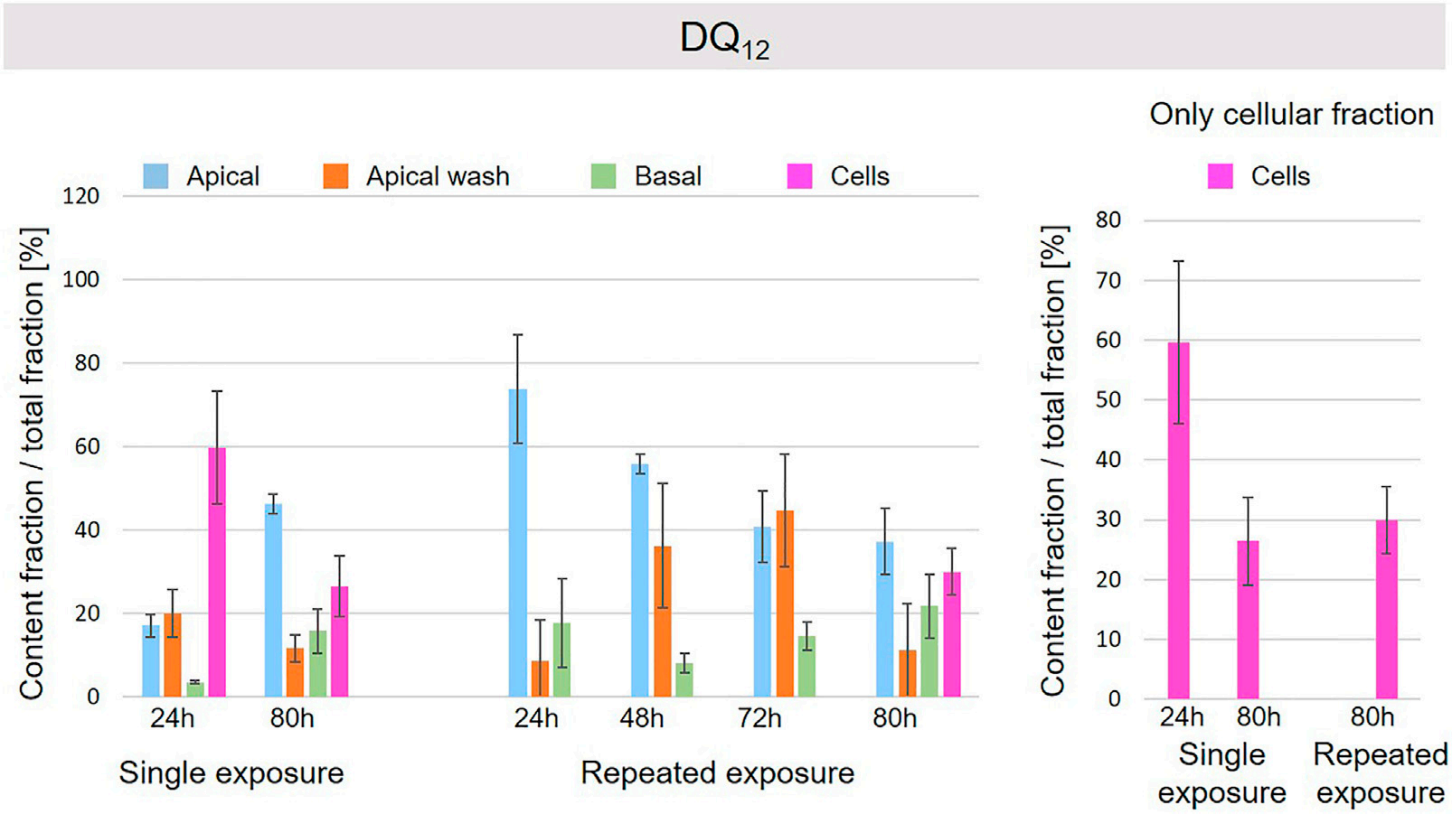
Fate of DQ_12_ at the A549 tissue barrier after single and repeated exposures. For single exposures DQ_12_ was exposed at a concentration of 100 µg/ml and the different fractions were collected after incubation for 24 and 80 h. For repeated exposures the cells were exposed repeatedly every day up to 80 h, using 25 µg/ml DQ_12_ concentration, resulting in a total exposure of 100 µg/ml and 80 h incubation time in total. Apical, apical cell wash and basal samples were collected after 24, 48, 72, and 80 h of exposure. Right side: This graph only depicts the cellular fraction. A total of three independent experiments (*n* = 3), with three single replicates each, were performed.

A schematic overview depicted in Figure 1 represents the experimental design of cell exposures and sampling. Single exposures were performed for all materials using 100 µg/ml and incubation for 24 and 80 h. After 24 and 80 h exposures, BaSO_4_ NMs (Figure 5), CeO_2_ NMs (Figure 6) and TiO_2_ NMs (Figure 7) were mainly found in the apical compartments with only a low amount in the basal ones. For ZnO NMs similar fractions were detected in the apical and basal compartments (Figure 8). The cell fraction was highest for BaSO_4_ NMs (30.3 ± 2.9%), then CeO_2_ NMs (13 ± 2.4%), while for TiO_2_ NMs (1.8 ± 1.3%) and ZnO NMs (4.4 ± 0.4%) the concentrations found were significantly lower. A different trend was observed for DQ_12_ (Figure 9), most of the material could be found in the cell fraction (59.7 ± 13.6%) whereas a lower amount was found in the apical and basal fraction. For all materials a decrease in the cellular fraction was observed from 24 to 80 h, for BaSO_4_ NMs from 30.3 ± 2.9% to 2 ± 0.1%, for CeO_2_ NMs from 13 ± 2.4% to 3 ± 0.8%, for TiO_2_ NMs from 1.8 ± 1.3% to 0.4 ± 0.4%, for ZnO NMs from 4.4 ± 0.4% to 2.3 ± 0.5%, and for DQ_12_ from 59.7 ± 13.6% to 26.4 ± 7.3%.

As far as repeated exposure experiments were done, as already shown for the single exposure experiments, BaSO_4_ NMs (Figure 5), CeO_2_ NMs (Figure 6) and TiO_2_ NMs (Figure 7) could be mainly detected in the apical fraction, and only a low concentration was detected in the basal fraction for all time points investigated. Again, for the ZnO NMs (Figure 8) samples, we detected similar concentrations in the apical and basal compartments at all time points. A different biodistribution pattern was observed for DQ_12_ (Figure 9). After the first 24 h, DQ_12_ was mainly detected in the apical compartment, and lower concentrations were also detected in the basal and apical wash samples. After 80 h, a clear decrease in the apical fraction was observed while the concentrations in the basal compartment was constant, but with a significant concentration of DQ12 the cell fraction. Expect for ZnO NMs, where no material was found inside cells after 80 h, all materials showed similar fractions in cells as measured for the single exposure and 80 h post-incubation, i.e., for BaSO_4_ NMs 7.9 ± 2.1% for CeO_2_ NMs 6%, for TiO_2_ NMs 1 ± 0.4%, and for DQ_12_ 26.9 ± 5.6%.

## Discussion

Structural and functional epithelial tissue barriers, e.g., skin, gastrointestinal or respiratory tract, are provided by the epithelia serving as an interface between biological compartments. The interaction of NMs with epithelia in an organism is a key aspect of hazard assessments as they act as barriers to the passage of materials into the organism (Rothen-Rutishauser et al., 2012). This includes the NM uptake by the cells and possible translocation of NMs across the tissue barriers. The aim of this study was to measure and quantify the translocation and the cellular uptake and attachment to the outer to the cell membrane of different (nano)materials (BaSO_4_ NMs, CeO_2_ NMs, DQ_12_, TiO_2_ NMs, ZnO NMs) across A549 epithelial lung cell monolayers by measuring the elemental distribution of the materials in different *in vitro* test compartments, e.g., apical, apical wash, intracellular, and basal.

In the present study, none of the materials caused any significant cytotoxicity after the exposure to NMs and DQ_12_ at 100 µg/ml for 80 h. A study performed with 1–50 μg/ml TiO_2_ for up to 2 months with A549 cells also reported no change in cell viability (Armand et al., 2016).

Considering the interaction and the fate of NMs at the A549 tissue barrier, some interesting trends based on the NMs nature were observed. ZnO NMs particles were barely detectable by HSI in A549 cells and were mainly found in the apical and basal compartments for both single and repeated exposure set-up. ZnO NMs are grouped as partially soluble material by DF4nanoGrouping (Landsiedel et al., 2017). In addition, it has been described that ZnO NMs can dissolve in lysosomal fluid and RPMI medium, especially in presence of BSA, and the dissolution rate is proportional to the exposure time (Reed et al., 2012; Keller et al., 2020a). Therefore, we suggest that in our cell culture conditions, i.e., RPMI + 10% FBS medium, 37°C, ZnO NMs easily solubilize and does not accumulate in cells as also described by others (Degen and Kosec, 2000; Fatehah et al., 2014; Zanoni et al., 2022). After 80 h of exposure, we assume that ZnO NMs or ions, internalized in cells and quickly dissolved, can translocate into the basal compartments. This can in principle occur by different ways such as exocytosis of Zn ions or by translocation of ions through the intercellular space. Likewise, for repeated exposure, ZnO NMs more likely dissolves and less particles interact with the cells’ layer, resulting in a negligible amount of ZnO NMs detected in the cell compartment by ICP-OES. However, this dilution of ZnO NMs could also be explained by cell division, exocytosis and/or possible dissolution in lysosomes.

Also, for TiO_2_ NMs only a minor fraction was detected in the cellular compartment and the major percentage of NMs was found in the apical compartment. It is suggested that TiO_2_ NMs is not delivered to the cellular surface and remains mainly in the apical cell medium compartment, thus only a small fraction of it is internalized by cells. In addition, a reduction of TiO_2_ NMs content in cells after a single exposure from 24 to 80 h is observed. As reported in previous works, TiO_2_ NMs slowly dissolve in lysosomal fluid (few loss percentages after 170 h in relevant media) (Keller et al., 2020a) and TiO_2_ NMs is classified as almost insoluble and active material by DF4nanoGrouping (Landsiedel et al., 2017). The decrease of intracellular TiO_2_ NMs could be due to cell division and/or loss of cells in the steady-state epithelial tissue. The repeated exposure to a lower TiO_2_NMs concentration resulted in a similar cellular fraction at 80 h as observed for the single exposure to a high concentration.

CeO_2_ and BaSO_4_ NMs presented a similar behavior. For both materials the major fraction was detected in the apical compartment, and a low fraction was found in the basal compartment. In addition, a significant fraction was observed in the cells after 24 h that was higher for BaSO_4_ than for CeO_2_ NMs and decreased for materials after 80 h to a similar content. The higher decrease for BaSO_4_ NMs can be explained by the different solubility of the materials in the lysosomal fluids. As reported in literature (Keller et al., 2020b), BaSO_4_ NMs dissolution rate is higher than for CeO_2_ NMs and supports our findings.

Finally, the highest intracellular content was found for DQ_12_ suggesting a faster delivery of the material to the cell surface in comparison to the NMs used here. In addition, a significant fraction was found in the basal compartment. It is reported in literature that DQ_12_, as well as in general silica nanoparticles, can slowly dissolve in media with a reduced pH value and in presence of high values of salinity (Gorrepati et al., 2010; Kumar et al., 2014; Choi and Kim, 2019). This behavior could explain the possible passage of silica through the cells’ layer into the basal compartment in form as ions if dissolved inside the cells.

## Conclusion

Understanding the fate of NM in cells is of high relevance as a possible accumulation at tissue barriers over repeated exposures to lower concentrations might result in similar effects as upon exposure to one high concentration. By robust and reproducible ICP-OES measurements it could be demonstrated that the fate highly depends on the material properties such as composition and solubility. If A549 cells are exposed once at high NMs concentration, a decrease in the cellular fraction from 24 to 80 h was observed for all materials, possibly due to different factors such as cell division, cell loss, exocytosis or ions excretions. This might be different for tissues where no cell division is occurring. Interestingly, a repeated low-dose exposure leads to the same intracellular NM burden as a single high dose after 80 h for all the materials with one exception for the only material grouped as partially soluble (ZnO NMs) that confirms the low tendency to accumulate within cell tissues. The experimental set-up proposed for sampling and detecting the elemental distribution in different *in vitro* test compartments at different exposure conditions, revealed the different translocation of NMs across epithelial tissues according to the NMs properties and occurring of phenomena such as cell division and/or loss of cells in the steady-state epithelial tissue. This allowed us to make mechanistic hypothesis on NMs interaction and translocation across cell tissues, improving our understanding of findings deriving by the long-term exposures of animals or humans to NMs.

## Data Availability

The raw data supporting the conclusions of this article will be made available by the authors, without undue reservation.
